# Does Governance Quality Matter for the Selection of Policy Stringency to Fight COVID-19?

**DOI:** 10.3390/ijerph19116679

**Published:** 2022-05-30

**Authors:** Yan Wang

**Affiliations:** School of Economics and Management, Huangshan University, Huangshan 245021, China; 79641@hsu.edu.cn

**Keywords:** governance quality, policy stringency, inverse U-shape, nonmonotonic, COVID-19

## Abstract

Independent of different national conditions, an indisputable fact is that the worldwide governments should play a role in fighting the ongoing COVID-19. To make clear the determinants of government response to tackle COVID-19, I investigate the impact of governance quality. To do so, I newly create an overall governance index based on six dimensions of Worldwide Governance Indicators (WGI) from the World Bank to proxy governance quality. I regress the overall governance index with controls on the stringency index from the Oxford COVID-19 Government Response Tracker database. Using pooled and panel data models with individual and time fixed effects, I find that the relationship between governance quality and policy stringency for 339 days across 163 countries is significantly nonmonotonic. Countries with middle governance quality select a high level of policy stringency in contrast to those with high and low governance quality. I also find that policy stringency significantly increases when daily new cases increase. The findings highlight the role of governance quality in deciding the stringency level of public health policies.

## 1. Introduction

“We are engaged as a country in a constant struggle to protect lives and livelihoods, and we must balance the restrictions we introduce against the long-term scars they leave, whether for business and jobs or our physical and mental health”, said the British Prime Minister Boris Johnson in a debate over another lockdown in December 2020 [1]. Not only the UK alone but also countries worldwide must quell this continuous COVID-19 pandemic. However, the level of policy stringency has varied across countries and over time. Some countries selected stringent health policies such as curfews or lockdowns that partially/completely banned people from moving around [2]. Others adopted lax health policies without banning people from going out and thus stopping their daily business [3]. Taking economic costs brought by stringent health policies into account, a low level is preferred [4,5]. Yet, a high level of policy stringency seems to be a better choice of life safety. The stringency level of policy thus becomes a dilemma for any country. Determining the determinant of the stringency level of policy helps a country tackle such a dilemma. In this study, I examine the impact of governance quality—the ability of a government to efficiently allocate limited resources and manage affairs at all administrative levels, on the choice of policy stringency.

I do so due to two reasons. First, in a pre-pandemic world, a large body of literature has shown that good governance acts decisively. For instance, good governance incents economic reforms [6], decreases tax evasion [7], increases debt relief [8], raises the environmental policy stringency [9], lowers the level of entrepreneurship [10], fosters equity and happiness [11], promotes socio-economic development [12], and lowers child mortality [13]. Nevertheless, detecting the causal impact of governance quality always relies on imperfect ways to deal with endogeneity. The COVID-19 pandemic provides a unique opportunity to overcome this issue because all governments, independent of their governance quality, are given a common challenge [14]. Hence, the case of COVID responses allows us to test a possible causal impact of governance quality on the way governments have addressed the challenges posed by the pandemic. Second, COVID-19-related studies have reported the impact of governance quality on the COVID-19-related disease outcomes rather than government responses. For instance, Nabin et al. [15], Baris and Pelizzo [16], and Liang et al. [17] found that governance quality deputized only by GOV helps to contain the COVID-19 spread and reduce COVID-19-related mortality. Even though Stojkoski et al. [18] included all six dimensions when explaining the COVID-19 disease outcomes, governance quality was treated as the control rather than the predictor. As “countries might simply differ in the accuracy with which they detect the infection” [19], it is difficult to avoid data subjectivity when reporting disease outcomes. Comparably, policy stringency—“information on different common policy responses that governments have taken to respond to the pandemic” [20]—is relatively objective to reflect government responses. However, whether governance quality plays a role in deciding the choice of policy stringency is unknown. I, thus, treat governance quality as the explanatory variable and policy stringency as the outcome variable to address the gap of no direct investigation between these two.

**Figure 1:**
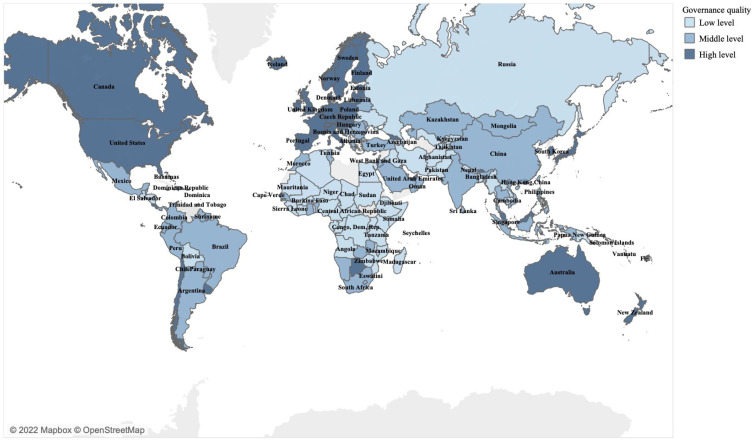
The 163 sample countries in this study. Notes: The mean of six WGI dimensions from WB decides the governance quality level.

To do so, I proxy governance quality by Worldwide Governance Indicators (WGI) from the World Bank (WB) (Worldwide governance indicators. At https://info.worldbank.org/governance/wgi/ (accessed on 21 September 2021)). WGI is “the best-known indicator” [21] to “track, measure, and rank broadly the quality of governance across time and countries” [16]. WB includes six WGI dimensions: “Voice and Accountability” (VOI), “Political Stability and Absence of Violence/Terrorism” (POL), “Government Effectiveness” (GOV), “Regulatory Quality” (REG), “Rule of Law” (RUL), and “Control of Corruption” (CON). To include the joint impact of these dimensions, I newly created overall governance indices to measure the level of governance quality. I merge governance quality data with a novel composite index that shows government policies’ intervention stringency [22] to fight COVID-19 from Oxford COVID-19 Government Response Tracker (Coronavirus Government Response Tracker. At https://www.bsg.ox.ac.uk/research/research-projects/coronavirus-government-response-tracker#data (accessed on 21 September 2021)) (hereafter: stringency index). The stringency index (SI) [23] is a composite index rescaled to a value from 0 (no measure) to 100 (completely lockdown) and updated in real-time. These countries in Figure 1 jointly represent over 95 percent of the global population (I use a population dataset provided by the World Bank. The total population in 2019 for 163 sample countries reached 7,492,067,121. At https://data.worldbank.org/indicator/SP.POP.TOTL?name_desc=true (accessed on 23 February 2022)) as of 2019. I take COVID-19 daily new cases from Our World in Data (OWID) to control the seriousness of COVID outbreak. Additionally, I control the heterogeneity of countries. The resulting dataset covers 339 days (from 28 January 2020 to 31 December 2020) for 163 countries. Due to the space limit, I report sample selection as Supplementary Materials (See Tables S1 and S2). As I focus on the first response of governments when facing a novel pandemic such as COVID-19, I select the year 2020 as the observing period. A year-long period also helps determine governmental ability to adjust measures when the pandemic develops from the first wave to the second one.

With pooled and panel data regression, I find that one standard deviation increase in governance quality decreases policy stringency by 0.08 to 2.19 standard deviations. Interestingly, governance quality and policy stringency follow a nonlinear relationship. Countries with middle governance quality select a high level of policy stringency relative to those with high and low governance quality. I also find that one standard deviation increase in daily new cases significantly increases policy stringency by 0.08 to 0.21 standard deviations. The two-way fixed effects model can capture the variation in the policy stringency up to 62%. The robustness checks are consistent with the main findings. I am the first to directly link governance quality with policymakers’ decisions in the context of COVID-19. I complement the extant studies that “good governance matters” [16]. The findings highlight the role of governance quality in deciding the stringency level of public health policies. 

The next section introduces the hypothesis. Section 3 outlines the construction of the datasets, including data collection and descriptive statistics, together with the model. Section 4 summarizes the main results. After further robustness checks in Section 5, I acknowledge the related literature and give discussions in Section 6. Finally, Section 7 draws conclusions and explain the limitations.

## 2. Hypothesis

Thus far, how stringent countries with various governance qualities respond to the COVID-19 pandemic is not that clear a priori. In my view, countries with different levels of governance quality consist of three groups: high-quality, middle-quality, and low-quality countries. WGI measures governance quality from three perspectives. Each perspective contains two dimensions: VOI and POL—the public participation in selecting, monitoring, and replacing governments; GOV and REG—the governmental effectiveness of formulating and implementing sound policies; RUL and CON—the public confidence in social rules and the integrity of public power [24]. Good governance is considered as wide public participation, high governmental efficiency, and full public confidence. To classify the sample countries, I make full use of six dimensions of WGI. In light of Friedman [10], I calculate the arithmetic mean of all six WGI dimensions (Mean_WGI). The joint impact of WGI helps avoid overlooking some WGI dimensions. I sort the value of Mean_WGI from the largest to the smallest for sample countries. Higher values of Mean_WGI are considered as higher governance quality. Relying on this rule, I group sample countries into three evenly. More details of the classification of countries are in Supplementary Materials (See Table S2). Figure 1 visualizes the level of governance quality across 163 sample countries. In this section, I expect the relationship between governance quality and policy stringency to fight COVID-19 follows a nonmonotonic tendency. Compared to countries with high and low governance quality, those with middle ones are more likely to impose a high level of policy stringency. This expectation relies on two perspectives:

The first is the capacity to fight the COVID-19 pandemic. In the logic of Fayissa and Nsiah [25], “governance directly impacts growth by determining how well the available resources work”. Countries with good governance quality are more likely to achieve good economic performance [26,27]—a factor critical to a country’s ability to provide economic relief packages and a robust healthcare system [28]. People tend to feel more confident when they can receive ample economic support and instant medical aid [29]. Hence, high-governance-quality countries are not in urgent need of a high level of policy stringency. Similarly, inspired by Licht et al. [30], Gaygısız [12] argued that “a country with ineffective and corrupt governance of institutions” fails to have good economic performance. In countries with low governance quality and poor economic performance, people run short of basic resources such as clean water, food, and the internet but are not limited to these resources. Now, stringent policies to fight COVID-19 have made things even worse. For instance, household income in rural Uganda dropped by 60% and household expenditure on food dropped by 40% [31]. In rural Nepal, lockdowns decreased working hours by 50% [32]. Lockdowns even killed people [33]. In Uganda, mothers in labor died amidst the COVID-19 lockdowns due to a ban on private transport and poorly available ambulance services [34]. In Africa, 23 economies have suspended measles vaccination campaigns as they have been coping with COVID-19 [35]. It is less likely for low-governance countries to afford stringent policies when people “have little way of surviving if they are forced indoors” [36]. 

The second is public compliance resulting from trust in governments. Governance quality allows “educated citizens complain more [37]” and thus promotes public trust in governments [38]. OECD [39] concludes that people show more compliance when they trust governments. Likewise, Bargain and Aminjonov [40] reported that “trust in governments is an important determinant of citizens’ compliance with public health policies” during the COVID-19 crisis. When people are “compatible” with governments, countries are more likely to achieve policy objectives [41]. Hence, the better the governance quality, the lower the policy stringency that governments tend to respond with. Notably, the COVID-19 pandemic also poses challenges to policing and security systems. Police forces are responsible for new public health regulations “without neglecting their traditional role of safeguarding” [42]. Low-governance-quality countries tend to have incompetent governments, which provide fragile policing and security institutions [43]. The incompetence of policing and security discourages trust in the police [44]. Lax policies tend to be more useful to avoid “harm to the vulnerable and the unrest in the country” [45] during the pandemic period. 

As mentioned above, economic performance is critical to available resources and medical systems for countries especially in the COVID-19 pandemic [46]. Relative to high/low-governance-quality peers, countries with middle governance quality tend to perform worse/better economically. Resources to support more restrictive policies for better social protection are less/more available to meet challenges posed by the COVID-19 pandemic. The medical system is likely to be less/more robust. Moreover, citizens tend to enjoy less/more freedom of complaint and governments tend to be less/more accountable. Overall, the capacity in middle-governance-quality countries to afford strict lockdowns is weaker/stronger than that in high/low ones. Hence, policy stringency is expected to be high in countries with middle governance quality than others.

## 3. Methodology

### 3.1. Data Collection

**Figure 2:**
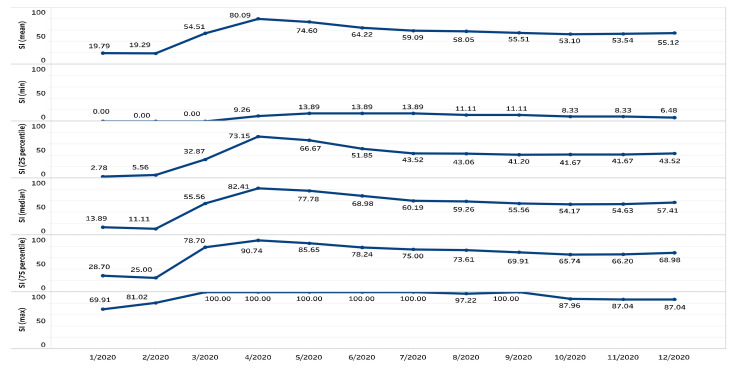
Variation in stringency index in 2020. Notes: I use policy stringency data from Oxford COVID-19 Government Response Tracker. Vertical axes show mean, minimum, 25 percentile, median, 75 percentile, and maximum of stringency index by month.

First, I follow Ashraf [3] and Frey et al. [47] to proxy policy stringency by the stringency index from Oxford COVID-19 Government Response Tracker (OxCGRT). The stringency index does not measure the appropriateness or effectiveness but the strictness of government responses such as school closures, workplace closures, and travel bans [22]. The lowest value of SI “0” represents no measure to fight the COVID-19, while the highest value “100“ represents complete lockdown. Governments act more strictly with a higher stringency index. I retrieved the stringency index data on 19 May 2021. The SI is at a country level and available for 185 countries. For all the 185 countries, these data start from 21 January 2020. I combine SI data with WGI datasets and additional controls that I present further down. As a result, I have a sample of 163 countries with 48,351 observations. To scrutinize how the impact of governance quality on the level of policy stringency varies over time, I separate SI observations by natural months. Hence, Table 1 reports descriptive statistics of policy stringency for the full sample and 12 monthly subsamples. Figure 2 visualizes these descriptive statistics. The trend of SI shows that the amount of stringency that governments have responded with to the COVID-19 pandemic is inverse U-shaped. To contain the COVID-19 spread, governments responded with different levels of stringency rather than a fixed level as time went on. Some countries increased the level of policy stringency to the highest level of SI (perfectly lockdown). Overall, SI tends to decrease.

I proxy my explanatory variable, governance quality, by WGI. WGI is country-level data from the WB used to compare governance quality across over 200 countries over 1996–2020. WGI measures governance quality based on six dimensions: VOI, POL, GOV, REG, RUL, and CON. The values of six WGI dimensions are in units of the standard normal distribution, with a mean of zero and standard deviation of one, ranging from approximately −2.5 to 2.5 [48]. Higher WGI values represent “better governance” [8] Table 2 confirms the significantly strong inter-correlations of six individual WGI dimensions. (The range of correlation is referred to in the article Correlation Coefficient: Simple Definition, Formula, Easy Steps. When the correlation coefficient is between 0.40 and 0.69, it means a strong positive relationship between the two variables, and the relationship is treated as very strong when the correlation coefficient is no less than 0.70. At https://www.statisticshowto.com/probability-and-statistics/correlation-coefficient-formula/ (accessed on 24 December 2020)) Due to a potential multicollinearity problem, I thus exclude introducing all six WGI dimensions into my model simultaneously but newly create a governance composite index. I follow Friedman [10] to use the arithmetic mean of all six WGI dimensions (Mean_WGI). This helps capture the joint impact of governance quality. I also create another two indices by Principal Component Analysis (hereafter: PCA_WGI) and Factor Analysis (hereafter: FA_WGI) for robustness checks. I list descriptive statistics of independent variables in Table 3. 

Table 3 also reports descriptive statistics of control variables. To represent the seriousness of the COVID-19 outbreak. I include COVID-19 disease outcomes. I follow Violato et al. [49] to proxy use daily new cases (smoothed per million) from OWID (the COVID-19 data maintained by OWID. At https://github.com/owid/covid-19-data/tree/master/public/data (accessed on 5 May 2022)). Daily new cases data are available for 209 countries from 28 January 2020 to 31 December 2020. The 1st COVID case was reported at a different time across countries and some countries fail to update data daily. I thus use the interpolation method to treat daily new cases. Due to some negative values of daily new cases, I use the Z-score to standardize these daily new cases to increase the accuracy of the results. After merging with SI datasets and other controls, the daily new cases see 48,351 observations for 163 countries. However, daily new cases can be manipulated by not-so-transparent governments who want to make the impression that the outbreak is smaller in their countries than it really is (by reducing testing). Daily new deaths data raise the same concern. I thus consider another alternative control—daily hospitalization for COVID seriousness. Daily hospitalization measures the amount of daily COVID-19 patients (per million) in hospital from OWID. Although it is only available for 34 countries, it helps control the robustness of the medical system in addition to the COVID-19 seriousness. 

As cross-country disparities are complex, I further include other country-level controls. As an economic control, GDP per capita is Gross Domestic Product converted to international dollars using purchasing power parity rates and divided by the total population [50] I retrieved 2019 GDP per capita by PPP Current international Dollars. The average GDP per capita is USD 23,330.00 for my sample countries. Additionally, I control for demographic indicators: population size and density due to their influence on the COVID-19 spread [16,51]. I obtain data in 2019 from the WB and also take natural logarithm values of these controls.

### 3.2. My Model

To scrutinize the relationship between governance quality and policy stringency, I construct the below function.

$$Policy\;stringency_{it}=\alpha_{i}+\beta_{1}Governance\;quality_{i}^{2}\;+\beta_{2}Governance\;quality_{i}+\gamma COVID_{it}+\delta Controls_{i}+\mu_{i}+\lambda_{t}+\xi_{it}\;$$

The dependent variable, policy stringency in the country *i* for a day *t*, is the function of governance quality. $\beta_{1}$ is the coefficient of Mean_WGI squared and $\beta_{2}$ is that of Mean_WGI. In both equations, I include daily new cases (smoothed per million) to proxy the seriousness of the COVID outbreak. As policy stringency in 2020 cannot impact GDP per capita, population, and population density in 2019, it is reasonable to include them as additional controls. In addition to COVID seriousness and other country-level controls, I introduce a series of individual and time dummy variables to apply country-specific ($\mu_{i}$) and time-specific ($\lambda_{t})$ effects. $\mu_{i\;}$ helps avoid invisible heterogeneity being omitted across countries, while $\lambda_{t}$ helps control changes caused by time trends. $\xi_{it}$ are standard errors. I treat the full dataset as pooled data and panel data. I run the Ordinary Least Squares Regression (OLS) model. In addition, the Least-squares Dummy Variable Regression (LSDV) model is applied when using country and month dummies to estimate the fixed effect [52].

## 4. Results

Table 4 reports regression results of the impact of governance quality on policy stringency. Columns (1)–(4) use pooled OLS regression and columns (5)–(8) use panel OLS regression. Except columns (1) and (5), I include country-specific or time-specific dummies or both in other columns. Standard errors are heteroskedasticity-robust. I use the standard βeta coefficient, which measures the standard deviation change in the response variable led by a standard deviation change in each predictor variable [52], to assess the impact of governance quality on the level of policy stringency. This helps to put all the regressors on a common base. 

**Figure 3:**
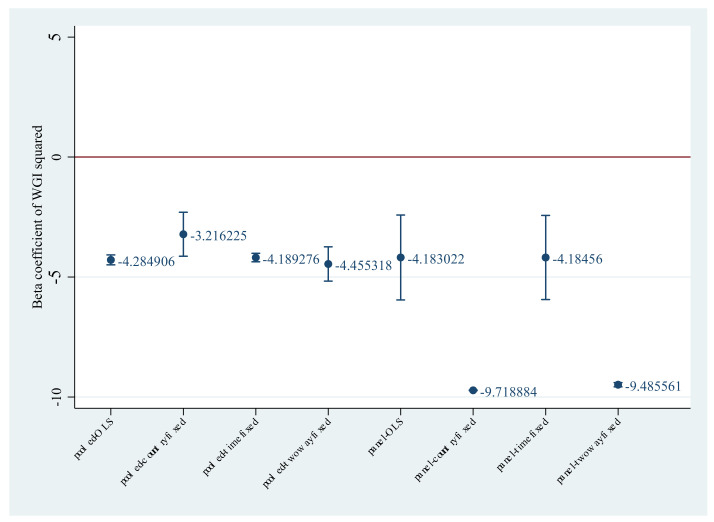
The beta coefficients of WGI squared. Notes: Horizontal axis is labeled as the regression models corresponding with columns (1) to (8) in Table 4. Vertical axis shows the value of beta coefficients of WGI squared.

Figure 3 visualizes the βeta coefficients of WGI squared. I focus on the sign of $\beta_{1}$ as it is crucial to test the hypothesis: whether the countries with middle governance quality impose a higher level of policy stringency than other peers. Compared with the reference line (WGI squared = 0), the negative sign of $\beta_{1}$ suggests that the relationship between governance quality and policy stringency is inverse U-shaped. It is consistent with the hypothesis at least for our sample. The standard βeta coefficient of $\beta_{2}$ ranges from −2.19 to −0.08. This means one standard deviation increase in governance quality decreases policy stringency by 0.08 to 2.19 standard deviations. Notably, daily new cases significantly increase policy stringency. To be concise, I report regression results based on hospitalization as Supplementary Materials (See Table S3). Daily COVID-19 patients in hospital also show a significantly positive impact on policy stringency. One standard deviation change in daily new cases significantly increases policy stringency by 0.08 to 0.21 standard deviations. Due to the space limit, I omit the beta coefficients of each country and each month.

For the goodness of fit, I report R^2^ in columns (1) to (4) but not adjusted R^2^, as I account for heteroscedasticity in the standard error term. The pooled data model can capture the variation in the policy stringency at 8% to 62%. In columns (5) to (8), I report R^2^_within, R^2^_between, and R^2^_overall. R^2^_between determines how much of the variation in policy stringency across sample countries is captured, while R^2^_within shows that within a country. Both pooled data and panel data regression show that the best prediction of the impact of governance quality relies on the two-way fixed effects. Interestingly, the variation in policy stringency can be explained at 40% when only controlling individual fixed effects. It is of 8% larger explanatory power compared with the only control of the time-fixed effect. It represents that the variation in policy stringency is influenced more by the heterogeneity of countries than that of time.

## 5. Further Robustness Checks

In this section, I check the robustness of the main findings above. First, I discard the extreme values of policy stringency and daily new cases data. To do so, I follow Neukirchen et al. [53] to winsor these two data at 1st and 99th percentiles and rerun all the models in Table 4. The observations decrease from 48,351 to 46,979. From the regression results in Table 5, the sign of WGI squared maintains consistency with the above main findings. One standard deviation change in governance quality decreases policy stringency up to 4.54 standard deviations. The explanatory power of the main model increases up to 64% of variations in policy stringency. It is also robust for winsorization at the 5th and 95th percentiles. The winsorization significantly improves the model results for my sample countries.

Second, I apply two weighted composite governance indices generated from Principal Component Analysis (hereafter: PCA_WGI) [12,18] and Factor Analysis (hereafter: FA_WGI) [25] to ensure the robustness of the overall governance index. Due to the space limit and better explanatory power of panel data regression, I check the impact of governance quality proxied by these two predictors with panel data. The regression results in Table 6 verify that countries with the middle governance quality level select a high level of policy stringency.

In addition, I substitute the overall governance index with one WGI dimension—“Government Effectiveness“. I follow Nabin et al. [15] to do so as “GOV captures the most generic aspects of quality of governance, whereas the other constituents capture more specific aspects”. Regression results in Table 7 also show strong evidence that the relationship between governance quality and policy stringency is inverse U-shaped.

## 6. Discussion

In this study, I find a significant nonmonotonic relationship between governance quality and policy stringency. Using an overall governance index as the predictor, middle-governance-quality countries implement a high level of policy stringency in contrast to other countries at least for my sample. Meanwhile, countries respond strictly when daily new cases increase, with this finding coinciding with Ma et al. [54] that governments’ responses are upgraded with the further spread of COVID-19. As I am the first to examine the link between governance quality and policy stringency, there are no direct points of comparison.

One possible explanation is related to that in Morita et al. [2] that developed countries are more burdened with the losses of convenience and economic opportunities owing to the behavior changes. According to the basic principle of economics that “People face tradeoffs” [55], raising the level of policy in response to COVID-19 is at the larger cost of economic activities. The opportunity cost of a one-day lockdown in developed countries, usually also well-governed, is different from that in developing countries. High-governance-quality countries are usually also developed countries. For the 163 sample countries in this paper, 54 high-governance countries consist of 47 high-income and 7 middle-income countries using the income grouping standard by WB. High-income countries have to give up more when implementing the same level of policy stringency compared to other countries. Consequently, rational governments are more likely to respond with a low level of policy stringency. In this sense, low-quality ones seem to have a small opportunity cost with a high level of policy stringency. However, a small opportunity cost does not mean that low-quality countries can select a low level of policy stringency. The tremendous reduction in economic activities caused by strict lockdowns make low-quality counties even worse off.

Another possible explanation is related to a country’s political system. Based on the 2019 Democracy index from the Economist, at least 22 countries are of full democracy and 21 of flawed democracy in my 54 good-governance-quality sample. None of the middle-and low-governance-quality countries are of full democracy but authoritarian and hybrid regimes. Thus far, there is no common knowledge whether democratic or authoritarian countries are better-performing. However, a government’s capacity in mobilizing scattered resources and organizing a large scale of speedy testing and tracing and even lockdowns is critical especially under the pandemic. Compared to democratic countries, authoritarian ones are more likely to reduce the time to make decisions, or buy time for vaccine and other containing tools. For low-governance countries, even with an authoritarian regime, poor economic performance still makes it difficult for them to have such a capacity. However, those of middle governance quality running with an authoritarian regime seem to be more advantageous.

An additional possible explanation relies on the public compliance. People have to trade some of their autonomy for compliance with government policies. Some middle-governance-quality countries start with introducing less stringent measures, but the population does not follow them properly, forcing these governments to be stricter. High-governance-quality countries get away with less stringent measures because the populations are cautious and follow restrictions. Low-governance-quality countries simply do not care.

## 7. Conclusions

To fight COVID-19, countries have implemented policies with different levels of stringency in response to the COVID-19 pandemic. Making the determinant of the level of policy stringency clear helps policymakers address their abilities for better decisions to fight COVID-19. In this study, I scrutinize the impact of governance quality on the cross-country variations in the policy stringency amid the COVID-19 pandemic. I find that governance quality significantly impacts policymakers’ decisions in a nonmonotonic way. Middle-governance-quality countries selected a higher level of policy stringency than other countries. In the absence of realistic models covering all related factors in explaining the variations in policy stringency, this study confirms the role of governance quality. 

To focus on the joint impact of governance quality, this study does not compare the impact of governance quality proxied by each individual governance indictor. Yet, as a proxy of governance quality, WGI is imperfect as it is difficult to observe the true level of governance in an economy [56]. Therefore, using the value of WGI composite index to group countries with different levels of governance quality may not be consistent with the real world. Additional indicators are expected in the future. Another issue to note is the difficult task when measuring public adherence to restrictions. Currently, vaccination is expected to come into effect for the virus spread control. However, the COVID virus continues mutating. Some mutations of the COVID virus are not only easier to spread than the original one but also escape antibodies after vaccination. Accordingly, more research is required compared to the determinants of policy stringency over the different waves of COVID-19 in the future.

## Figures and Tables

**Table 1:** Descriptive statistics of the outcome variable.

Variable	N	Mean	St. Dev.	Minimum	25th Percentile	Median	75th Percentile	Maximum
policy stringency (Full sample)	48,351	60.22	21.16	0.00	45.37	62.50	77.31	100.00
2020								
January	45	19.79	21.97	0.00	2.78	13.89	28.70	69.91
February	775	19.29	18.93	0.00	5.56	11.11	25	81.02
March	3233	54.51	27.52	0.00	32.87	55.56	78.70	100.00
April	4753	80.09	14.89	9.26	73.15	82.41	90.74	100.00
May	4969	74.60	15.69	13.89	66.67	77.78	85.65	100.00
June	4830	64.22	18.38	13.89	51.85	68.98	78.24	100.00
July	4991	59.09	19.35	13.89	43.52	60.19	75	100.00
August	4991	58.05	19.11	11.11	43.06	59.26	73.61	97.22
September	4830	55.51	18.47	11.11	41.20	55.56	69.91	100.00
October	5006	53.10	16.90	8.33	41.67	54.17	65.74	87.96
November	4875	53.54	17.08	8.33	41.67	54.63	66.20	87.04
December	5053	55.12	17.85	6.48	43.52	57.41	68.98	87.04

Notes: I use policy stringency data from Oxford COVID-19 Government Response Tracker. Calculated by Stata 17.0.

**Table 2:** Correlation matrix of all six WGI dimensions for 163 sample countries.

Variable	VOI	POL	GOV	REG	RUL	CON	Mean_WGI	PCA_WGI	FA_WGI
VOI	1								
POL	0.67 ***	1							
GOV	0.68 ***	0.75 ***	1						
REG	0.73 ***	0.72 ***	0.95 ***	1					
RUL	0.75 ***	0.78 ***	0.95 ***	0.93 ***	1				
CON	0.74 ***	0.77 ***	0.92 ***	0.88 ***	0.94 ***	1			
Mean_WGI	0.83 ***	0.85 ***	0.95 ***	0.95 ***	0.97 ***	0.96 ***	1		
PCA_WGI	0.82 ***	0.85 ***	0.96 ***	0.95 ***	0.97 ***	0.96 ***	0.99 ***	1	
FA_WGI	0.78 ***	0.81 ***	0.97 ***	0.96 ***	0.99 ***	0.96 ***	0.99 ***	0.99 ***	1

Notes: *** *p* < 0.01. Worldwide Governance Indicators (WGI) come from the World Bank. Mean_WGI is the mean of all six WGI dimensions. PCA_WGI is a composite WGI index created by Principal Component Analysis. FA_WGI is a composite WGI index created by Factor Analysis. VOI, POL, GOV, REG, RUL, and CON represent “Voice and Accountability”, “Political Stability and Absence of Violence/Terrorism”, “Government Effectiveness”, “Regulatory Quality”, “Rule of Law”, and “Control of Corruption” individually. Calculated by Stata 17.0.

**Table 3:** Descriptive statistics of explanatory and control variables.

Variable	N	Mean	St. Dev.	Minimum	25th Percentile	50th Percentile	75th Percentile	Maximum
Mean_WGI	163	0.00	0.88	−1.61	−0.66	−0.17	0.65	1.77
PCA_WGI	163	0.01	2.26	−4.13	−1.69	−0.44	1.68	4.58
FA_WGI	163	0.00	0.99	−1.79	−0.72	−0.21	0.70	2.04
Daily new cases per million	48,351	51.09	119.09	0.00	0.79	6.05	42.85	634.83
Daily hospitalization per million	9092	118.82	182.61	0	11.93	44.80	849.11	1416.13
GDP per capita	163	23,330.00	24,210.00	980.30	4955.00	13,635.00	36,945.00	119,416.00
Population (million)	163	45.96	157.43	0.07	3.59	10.28	33.92	1397.72
Population density	163	366.40	1735.00	3.03	30.52	82.22	197.60	8045.00

Notes: Mean_WGI is the mean of all six WGI dimensions. PCA_WGI is a composite WGI index by using Principal Component Analysis. FA_WGI is a composite WGI index by using Factor Analysis. Worldwide Governance Indicators (WGI, 2019) are from the World Bank. GDP per capita (2019), population (2019), and population density (2018) come from the World Bank Development Indicator. Mean years of schooling and life expectancy at birth are from the United Nations Development Program. Calculated by Stata 17.0.

**Table 4:** Regression results of the main model.

	Pooled Data				Panel Data			
	(1) OLS	(2) LSDV	(3) LSDV	(4) LSDV	(1) OLS	(2) LSDV	(3) LSDV	(4) LSDV
Mean_WGI squared	−0.18 ***	−0.14 ***	−0.18 ***	−0.19 ***	−0.18 ***	−0.41 ***	−0.18 ***	−0.40 ***
	(0.11)	(0.47)	(0.09)	(0.37)	(0.90)	(0.01)	(0.89)	(0.05)
Mean_WGI	−0.20 ***	−0.08 **	−0.19 ***	−0.01	−0.23 ***	−2.19 ***	−0.21 ***	−2.18 ***
	(0.19)	(0.85)	(0.17)	(0.66)	(2.05)	(0.07)	(1.98)	(0.15)
Daily new cases	0.12 ***	0.08 ***	0.23 ***	0.21 ***	0.08 ***	0.08 ***	0.21 ***	0.21 ***
	(0.08)	(0.07)	(0.10)	(0.08)	(0.40)	(0.40)	(0.49)	(0.49)
GDP per capita	0.20 ***	0.20 ***	0.18 ***	0.09 ***	0.25 ***	2.90 ***	0.21 **	2.92 ***
	(0.14)	(0.36)	(0.12)	(0.26)	(1.62)	(0.11)	(1.55)	(0.15)
Population	0.10 ***	0.45 ***	0.11 ***	0.38 ***	0.11 **	−0.48 ***	0.12 ***	−0.40 ***
	(0.05)	(0.17)	(0.04)	(0.14)	(0.53)	(0.07)	(0.50)	(0.11)
Population density	−0.07 ***	−0.14 ***	−0.06 ***	−0.13 ***	−0.06	−0.41 ***	−0.06	−0.42 ***
	(0.06)	(0.17)	(0.05)	(0.13)	(0.69)	(0.07)	(0.66)	(0.12)
Individual fixed effect	No	Yes	No	Yes	No	Yes	No	Yes
Time fixed effect	No	No	Yes	Yes	No	No	Yes	Yes
Observations	48,351	48,351	48,351	48,351	48,351	48,351	48,351	48,351
R^2^	0.08	0.40	0.32	0.62				
R^2^_overall					0.08	0.40	0.32	0.62
R^2^_between					0.21	1.00	0.25	1.00
R^2^_within					0.01	0.01	0.37	0.37

Notes: In all columns, I report standard beta coefficients and corresponding significance (** *p* < 0.05, *** *p* < 0.01). Heteroskedasticity-robust standard errors in parentheses. Governance quality is measured by Mean_WGI: the mean of all six WGI dimensions. Calculated by Stata 17.0.

**Table 5:** Robustness checks: winsorization.

	Pooled Data				Panel Data			
	OLS	LSDV	LSDV	LSDV	OLS	LSDV	LSDV	LSDV
Mean_WGI squared	−0.18 ***	−0.07 ***	−0.17 ***	−0.13 ***	−0.17 ***	−2.59 ***	−0.17 ***	−2.29 ***
	(0.10)	(0.47)	(0.09)	(0.37)	(0.89)	(0.03)	(0.89)	(0.78)
Mean_WGI	−0.19 ***	0.01	−0.19 ***	0.08 ***	−0.22 **	−4.54 ***	−0.21 **	−3.92 ***
	(0.19)	(0.71)	(0.16)	(0.54)	(2.05)	(0.22)	(1.96)	(1.31)
Daily new cases	0.12 ***	0.06 ***	0.25 ***	0.22 ***	0.06 ***	0.06 ***	0.22 ***	0.22 ***
	(0.10)	(0.10)	(0.11)	(0.10)	(0.54)	(0.55)	(0.60)	(0.61)
GDP per capita	0.20 ***	0.22 ***	0.16 ***	0.09 ***	0.26 ***	0.50 ***	0.20 **	0.55 ***
	(0.14)	(0.37)	(0.12)	(0.27)	(1.61)	(0.04)	(1.54)	(0.49)
Population	0.12 ***	0.46 ***	0.13 ***	0.39 ***	0.14 ***	−0.12 ***	0.13 ***	−0.13 ***
	(0.05)	(0.16)	(0.04)	(0.14)	(0.52)	(0.07)	(0.49)	(0.13)
Population density	−0.08 ***	−0.18 ***	−0.08 ***	−0.18 ***	−0.08	−0.23 ***	−0.07	−0.33 ***
	(0.06)	(0.14)	(0.05)	(0.11)	(0.69)	(0.12)	(0.66)	(0.19)
Country fixed effect	No	Yes	No	Yes	No	Yes	No	Yes
Time fixed effect	No	No	Yes	Yes	No	No	Yes	Yes
Observations	46,979	46,979	46,979	46,979	46,979	46,979	46,979	46,979
R^2^	0.09	0.42	0.32	0.64				
R^2^_overall					0.09	0.42	0.32	0.64
R^2^_between					0.21	1.00	0.25	1.00
R^2^_within					0.00	0.00	0.38	0.38

Notes: In all columns, I report standard beta coefficients and corresponding significance (** *p* < 0.05, *** *p* < 0.01). Heteroskedasticity-robust standard errors in parentheses. Governance quality is measured by Mean_WGI: the mean of all six WGI dimensions. Calculated by Stata 17.0.

**Table 6:** Robustness checks: new overall governance indices.

	Panel Data				Panel Data			
	OLS	LSDV	LSDV	LSDV	OLS	LSDV	LSDV	LSDV
PCA_WGI squared	−0.17 ***	−2.79 ***	−0.17 ***	−2.47 ***				
	(0.13)	(0.00)	(0.13)	(0.13)				
FA_WGI squared					−0.16 ***	−0.49 ***	−0.16 ***	−0.48 ***
					(0.72)	(0.03)	(0.72)	(0.03)
PCA_WGI	−0.22 **	−4.77 ***	−0.21 **	−4.11 ***				
	(0.80)	(0.09)	(0.77)	(0.53)				
FA_WGI					−0.24 **	−2.77 ***	−0.22 **	−2.39 ***
					(2.01)	(0.22)	(1.94)	(0.62)
Daily new cases	0.06 ***	0.06 ***	0.22 ***	0.22 ***	0.06 ***	0.06 ***	0.22 ***	0.22 ***
	(0.54)	(0.55)	(0.60)	(0.61)	(0.54)	(0.55)	(0.60)	(0.61)
GDP per capita	0.27 ***	0.43 ***	0.20 **	0.49 ***	0.28 ***	3.28 ***	0.21 **	2.98 ***
	(1.63)	(0.04)	(1.55)	(0.50)	(1.74)	(0.04)	(1.67)	(0.41)
Population	0.14 ***	−0.07 ***	0.13 ***	−0.09 ***	0.14 ***	−0.43 ***	0.14 ***	−0.39 ***
	(0.52)	(0.07)	(0.49)	(0.14)	(0.51)	(0.07)	(0.48)	(0.10)
Population density	−0.07	−0.22 ***	−0.07	−0.32 ***	−0.07	−0.06 ***	−0.06	−0.20 ***
	(0.69)	(0.12)	(0.66)	(0.19)	(0.70)	(0.14)	(0.67)	(0.21)
Country fixed effect	No	Yes	No	Yes	No	Yes	No	Yes
Time fixed effect	No	No	Yes	Yes	No	No	Yes	Yes
Observations	46,979	46,979	46,979	46,979	46,979	46,979	46,979	46,979
R^2^_overall	0.09	0.42	0.32	0.64	0.09	0.42	0.32	0.64
R^2^_between	0.21	1.00	0.25	1.00	0.21	1.00	0.24	1.00
R^2^_within	0.00	0.00	0.38	0.38	0.00	0.00	0.38	0.38

Notes: In all columns, I report standard beta coefficients and corresponding significance (** *p* < 0.05, *** *p* < 0.01). Heteroskedasticity-robust standard errors in parentheses. Governance quality is measured by PCA_WGI and FA_WGI. PCA_WGI is a composite WGI index created by Principal Component Analysis. FA_WGI is a composite WGI index created by Factor Analysis. Calculated by Stata 17.0.

**Table 7:** Robustness checks: one WGI dimension.

	Pooled Data				Panel Data			
	OLS	LSDV	LSDV	LSDV	OLS	LSDV	LSDV	LSDV
GOV squared	−0.16 ***	−0.09 ***	−0.15 ***	−0.09 ***	−0.16 ***	−0.46 ***	−0.15 ***	−0.45 ***
	(0.09)	(0.32)	(0.07)	(0.24)	(0.69)	(0.02)	(0.70)	(0.02)
GOV	−0.28 ***	0.71 ***	−0.27 ***	0.68 ***	−0.30 ***	−0.69 ***	−0.28 ***	−0.57 ***
	(0.20)	(0.83)	(0.17)	(0.65)	(2.04)	(0.12)	(1.98)	(0.29)
Daily new cases	0.11 ***	0.08 ***	0.22 ***	0.21 ***	0.08 ***	0.08 ***	0.21 ***	0.21 ***
	(0.08)	(0.07)	(0.10)	(0.08)	(0.40)	(0.40)	(0.49)	(0.49)
GDP per capita	0.28 ***	−0.63 ***	0.25 ***	−0.61 ***	0.32 ***	1.53 ***	0.28 ***	1.42 ***
	(0.16)	(0.43)	(0.15)	(0.37)	(1.91)	(0.02)	(1.86)	(0.28)
Population	0.12 ***	0.22 ***	0.14 ***	0.23 ***	0.14 ***	−0.64 ***	0.14 ***	−0.59 ***
	(0.05)	(0.24)	(0.04)	(0.20)	(0.50)	(0.04)	(0.46)	(0.06)
Population density	−0.04 ***	−0.02 ***	−0.03 ***	−0.01	−0.03	−0.55 ***	−0.03	−0.56 ***
	(0.06)	(0.10)	(0.05)	(0.11)	(0.69)	(0.09)	(0.67)	(0.12)
Country fixed effect	No	Yes	No	Yes	No	Yes	No	Yes
Time fixed effect	No	No	Yes	Yes	No	No	Yes	Yes
Observations	48,351	48,351	48,351	48,351	48,351	48,351	48,351	48,351
R^2^	0.08	0.40	0.31	0.62				
R^2^_overall					0.08	0.40	0.31	0.62
R^2^_between					0.21	1.00	0.24	1.00
R^2^_within					0.01	0.01	0.37	0.37

Notes: In all columns, I report standard beta coefficients and corresponding significance (*** *p* < 0.01). Heteroskedasticity-robust standard errors in parentheses. Governance quality is measured by “Government Effectiveness” (GOV). Calculated by Stata 17.0.

## Data Availability

All data available on request.

## Supplementary Materials

The following supporting information can be downloaded at: https://www.mdpi.com/article/10.3390/ijerph19116679/s1, Table S1: Sample selection, Table S2: The list of sample countries, Table S3: Regression results based on an alternative control of COVID seriousness.
