# Supplementary material for: Does Governance Quality Matter for the Selection of Policy Stringency to Fight COVID-19?
Source: Int J Environ Res Public Health. 2022 May 30;19(11):6679. doi: 10.3390/ijerph19116679 (PMC9180495; doi:10.3390/ijerph19116679)
Supplement: Supplementary file 1 [file ijerph-19-06679-s001.zip › ijerph-1697896-supplementary.pdf]

Supplementary Materials

Table S1 Sample selection

Variable	Source	Proxy	Countries included	Countries excluded	Remark
Dependent	OxCGRT	Stringency index (SI)	185		Original sample basis
Independent	WB	Worldwide Governance Indicator (WGI)		-1	x1 (Faeroe Islands)
				-5	x2 (U. S. Virgin Islands, Bermuda, Guam, Monaco, San Marino)
Control	WB	GDP per capita		-10	x3 (Andorra, Cuba, Eritrea, Greenland, Liechtenstein, South Sudan, Syria, Taiwan, Venezuela, Yemen)
Control	WB	Population			
Control	WB	Population density		-1	x4(Kosovo)
Control	OWID	Daily new cases		-4	x5 (Kiribati, Puerto Rico, Tonga, Turkmenistan)
				-1	x6 (Libya)
	Sample countries		163		See in Table S2

Notes: OxCGRT, WB and UNDP are the short for Oxford COVID-19 Government Response Tracker, the World Bank and United Nations Development. Program individually. 'x1' represents countries missing values of all six WGI dimensions. 'x2' represents countries missing values of part of WGI dimensions. 'x3' represents countries missing the 2019 GDP per capita values. 'x4' represents countries missing the 2019 population density values. 'x5' represents countries missing daily new cases. I also drop Libya due to the war situation.

Table S2 The list of sample countries

No.	Continent	Country	Mean_WGI	PCA_WGI	FA_WGI	Governance quality
1	Asia	Afghanistan	-1.56	-3.98	-1.66	Low level
2	Europe	Albania	-0.08	-0.20	-0.21	Low level
3	Africa	Algeria	-0.88	-2.25	-0.92	Low level
4	Africa	Angola	-0.87	-2.25	-1.07	Low level
5	South America	Argentina	-0.10	-0.29	-0.26	Middle level
6	South America	Aruba	1.17	3.00	1.25	High level
7	Oceania	Australia	1.57	4.07	1.82	High level
8	Europe	Austria	1.45	3.76	1.73	High level
9	Asia	Azerbaijan	-0.66	-1.64	-0.61	Low level
10	North America	Bahamas	0.58	1.45	0.45	High level
11	Asia	Bahrain	-0.13	-0.23	0.19	Middle level
12	Asia	Bangladesh	-0.82	-2.11	-0.87	Low level
13	North America	Barbados	0.79	2.00	0.71	High level
14	Europe	Belarus	-0.45	-1.13	-0.54	Middle level
15	Europe	Belgium	1.18	3.06	1.38	High level
16	North America	Belize	-0.27	-0.74	-0.53	Middle level
17	Africa	Benin	-0.34	-0.90	-0.49	Middle level
18	Asia	Bhutan	0.56	1.45	0.60	High level
19	South America	Bolivia	-0.71	-1.86	-0.95	Low level
20	Europe	Bosnia and Herzegovina	-0.38	-0.97	-0.41	Middle level
21	Africa	Botswana	0.59	1.51	0.57	High level
22	South America	Brazil	-0.18	-0.47	-0.22	Middle level
23	Asia	Brunei	0.60	1.61	0.76	High level
24	Europe	Bulgaria	0.28	0.72	0.20	Middle level
25	Africa	Burkina Faso	-0.52	-1.32	-0.51	Low level
26	Africa	Burundi	-1.42	-3.61	-1.49	Low level
27	Asia	Cambodia	-0.78	-1.99	-0.93	Low level
28	Africa	Cameroon	-1.12	-2.84	-1.16	Low level
29	North America	Canada	1.58	4.10	1.83	High level
30	Africa	Cape Verde	0.55	1.38	0.49	High level
31	Africa	Central African Republic	-1.58	-4.05	-1.72	Low level
32	Africa	Chad	-1.36	-3.48	-1.47	Low level
33	South America	Chile	0.95	2.47	1.13	High level
34	Asia	China	-0.36	-0.86	-0.25	Middle level
35	South America	Colombia	-0.14	-0.34	-0.16	Middle level
36	Africa	Congo	-1.23	-3.17	-1.37	Low level
37	Africa	Congo, Dem. Rep.	-1.61	-4.13	-1.79	Low level
38	North America	Costa Rica	0.63	1.60	0.62	High level
39	Africa	Cote d'Ivoire	-0.50	-1.27	-0.54	Low level
40	Europe	Croatia	0.46	1.19	0.43	High level
41	Europe	Cyprus	0.83	2.14	0.89	High level
42	Europe	Czech Republic	0.93	2.41	1.02	High level
43	Europe	Denmark	1.68	4.36	1.97	High level
44	Africa	Djibouti	-0.83	-2.12	-0.93	Low level
45	North America	Dominica	0.50	1.26	0.46	High level
46	North America	Dominican Republic	-0.22	-0.57	-0.35	Middle level
47	South America	Ecuador	-0.40	-1.07	-0.56	Middle level
48	Africa	Egypt	-0.81	-2.02	-0.70	Low level
49	North America	El Salvador	-0.29	-0.77	-0.49	Middle level
50	Europe	Estonia	1.24	3.22	1.43	High level
51	Africa	Eswatini	-0.66	-1.67	-0.66	Low level
52	Africa	Ethiopia	-0.79	-1.99	-0.71	Low level
53	Oceania	Fiji	0.25	0.62	0.15	Middle level
54	Europe	Finland	1.74	4.53	2.07	High level
55	Europe	France	1.17	3.04	1.42	High level
56	Africa	Gabon	-0.79	-2.03	-0.91	Low level
57	Africa	Gambia	-0.34	-0.90	-0.46	Middle level
58	Asia	Georgia	0.45	1.21	0.60	High level
59	Europe	Germany	1.46	3.80	1.74	High level
60	Africa	Ghana	0.05	0.12	-0.02	Middle level
61	Europe	Greece	0.39	1.00	0.33	Middle level
62	North America	Guatemala	-0.62	-1.60	-0.81	Low level

63	Africa	Guinea	-0.87	-2.25	-1.04	Low level
64	South America	Guyana	-0.25	-0.66	-0.38	Middle level
65	North America	Haiti	-1.18	-3.06	-1.36	Low level
66	North America	Honduras	-0.67	-1.72	-0.83	Low level
67	Asia	Hong Kong,China	1.16	3.10	1.64	High level
68	Europe	Hungary	0.42	1.11	0.45	High level
69	Europe	Iceland	1.56	4.03	1.75	High level
70	Asia	India	-0.11	-0.27	-0.08	Middle level
71	Asia	Indonesia	-0.17	-0.42	-0.22	Middle level
72	Asia	Iran	-1.14	-2.89	-1.07	Low level
73	Asia	Iraq	-1.51	-3.88	-1.63	Low level
74	Europe	Ireland	1.34	3.47	1.51	High level
75	Asia	Israel	0.73	1.95	1.05	High level
76	Europe	Italy	0.56	1.44	0.51	High level
77	North America	Jamaica	0.22	0.56	0.06	Middle level
78	Asia	Japan	1.32	3.44	1.55	High level
79	Asia	Jordan	-0.10	-0.21	0.03	Middle level
80	Asia	Kazakhstan	-0.30	-0.71	-0.28	Middle level
81	Africa	Kenya	-0.55	-1.39	-0.55	Low level
82	Asia	Kuwait	-0.04	-0.08	0.03	Middle level
83	Asia	Kyrgyzstan	-0.62	-1.60	-0.78	Low level
84	Asia	Laos	-0.80	-2.03	-0.95	Low level
85	Europe	Latvia	0.86	2.23	1.00	High level
86	Asia	Lebanon	-0.90	-2.29	-0.93	Low level
87	Africa	Lesotho	-0.35	-0.92	-0.44	Middle level
88	Africa	Liberia	-0.75	-1.97	-1.02	Low level
89	Europe	Lithuania	0.96	2.49	1.06	High level
90	Europe	Luxembourg	1.70	4.41	1.93	High level
91	Asia	Macau,China	0.89	2.37	1.07	High level
92	Africa	Madagascar	-0.73	-1.90	-0.98	Low level
93	Africa	Malawi	-0.49	-1.27	-0.58	Low level
94	Asia	Malaysia	0.43	1.16	0.59	High level
95	Africa	Mali	-0.95	-2.43	-0.93	Low level
96	Europe	Malta	0.87	2.23	0.89	High level
97	Africa	Mauritania	-0.67	-1.71	-0.72	Low level
98	Africa	Mauritius	0.76	1.98	0.81	High level
99	North America	Mexico	-0.37	-0.95	-0.47	Middle level
100	Europe	Moldova	-0.31	-0.78	-0.37	Middle level
101	Asia	Mongolia	0.01	0.01	-0.17	Middle level
102	Africa	Morocco	-0.29	-0.71	-0.24	Middle level
103	Africa	Mozambique	-0.77	-1.99	-0.93	Low level
104	Asia	Myanmar	-0.95	-2.43	-1.03	Low level
105	Africa	Namibia	0.29	0.73	0.25	Middle level
106	Asia	Nepal	-0.59	-1.54	-0.71	Low level
107	Europe	Netherlands	1.65	4.28	1.93	High level
108	Oceania	New Zealand	1.78	4.61	2.01	High level
109	North America	Nicaragua	-0.98	-2.49	-1.09	Low level
110	Africa	Niger	-0.75	-1.91	-0.72	Low level
111	Africa	Nigeria	-1.05	-2.68	-1.08	Low level
112	Europe	Norway	1.77	4.58	2.04	High level
113	Asia	Oman	0.17	0.50	0.35	Middle level
114	Asia	Pakistan	-0.99	-2.49	-0.87	Low level
115	North America	Panama	0.11	0.27	-0.02	Middle level
116	Oceania	Papua New Guinea	-0.65	-1.69	-0.81	Low level
117	South America	Paraguay	-0.34	-0.90	-0.52	Middle level
118	South America	Peru	-0.05	-0.12	-0.18	Middle level
119	Asia	Philippines	-0.31	-0.77	-0.34	Middle level
120	Europe	Poland	0.65	1.68	0.67	High level
121	Europe	Portugal	1.07	2.74	1.14	High level
122	Asia	Qatar	0.40	1.11	0.65	Middle level
123	Europe	Romania	0.24	0.61	0.21	Middle level
124	Europe	Russia	-0.58	-1.45	-0.61	Low level
125	Asia	Rwanda	-0.01	0.03	0.11	Middle level
126	Asia	Saudi Arabia	-0.23	-0.49	0.02	Middle level
127	Africa	Senegal	0.00	-0.01	-0.08	Middle level
128	Europe	Serbia	-0.08	-0.20	-0.12	Middle level

129	Africa	Seychelles	0.42	1.08	0.38	High level
130	Africa	Sierra Leone	-0.56	-1.49	-0.78	Low level
131	Asia	Singapore	1.63	4.30	2.04	High level
132	Europe	Slovakia	0.71	1.83	0.70	High level
133	Europe	Slovenia	0.99	2.56	1.12	High level
134	Oceania	Solomon Islands	-0.15	-0.44	-0.34	Middle level
135	Africa	Somalia	-2.12	-5.45	-2.36	Low level
136	Africa	South Africa	0.16	0.42	0.11	Middle level
137	Asia	South Korea	0.94	2.46	1.15	High level
138	Europe	Spain	0.85	2.20	0.98	High level
139	Asia	Sri Lanka	-0.15	-0.38	-0.15	Middle level
140	Africa	Sudan	-1.52	-3.88	-1.55	Low level
141	South America	Suriname	-0.20	-0.56	-0.31	Middle level
142	Europe	Sweden	1.72	4.45	2.00	High level
143	Europe	Switzerland	1.73	4.48	1.98	High level
144	Asia	Tajikistan	-1.17	-2.98	-1.29	Low level
145	Africa	Tanzania	-0.56	-1.44	-0.65	Low level
146	Asia	Thailand	-0.20	-0.45	-0.05	Middle level
147	Asia	Timor	-0.42	-1.16	-0.78	Middle level
148	Africa	Togo	-0.74	-1.90	-0.78	Low level
149	North America	Trinidad and Tobago	0.05	0.11	-0.05	Middle level
150	Africa	Tunisia	-0.18	-0.47	-0.13	Middle level
151	Asia	Turkey	-0.45	-1.09	-0.30	Middle level
152	Africa	Uganda	-0.62	-1.57	-0.62	Low level
153	Europe	Ukraine	-0.57	-1.46	-0.61	Low level
154	Asia	United Arab Emirates	0.65	1.77	0.94	High level
155	Europe	United Kingdom	1.37	3.57	1.65	High level
156	North America	United States	1.13	2.96	1.41	High level
157	South America	Uruguay	0.90	2.28	0.85	High level
158	Asia	Uzbekistan	-0.91	-2.33	-1.03	Low level
159	Oceania	Vanuatu	0.10	0.20	-0.07	Middle level
160	Asia	Vietnam	-0.33	-0.80	-0.24	Middle level
161	Asia	West Bank and Gaza	-0.73	-1.81	-0.57	Low level
162	Africa	Zambia	-0.45	-1.18	-0.57	Middle level
163	Africa	Zimbabwe	-1.20	-3.10	-1.37	Low level

Notes: I newly create three WGI composite indices based on all six dimensions of WGI from the WB. Mean_WGI is the mean of all six WGI dimensions. PCA_WGI is a composite WGI index by using Principal Component Analysis. FA_WGI is a composite WGI index by using Factor Analysis. I rank the level of governance quality based on the value of Mean_WGI. Notably, Qatar is considered as a high-level governance quality country rather than a middle-level one when using PCA_WGI and FA_WGI to substitute WGI. Opposite to Qatar, Seychelles is a high-level one when using Mean_WGI but a middle-level one with PCA_WGI or FA_WGI. Additionally, three countries (Ecuador, Timor, Zambia) are those with low governance quality by FA_WGI while middle by Mean_WGI or PCA_WGI. Another three (Burkina Faso, Cote d'Ivoire, Kenya) are those with middle governance quality by FA_WGI while low by Mean_WGI or PCA_WGI.

Table S3 Regression results of the main model

	Pooled data				Panel data			
	(1)	(2)	(3)	(4)	(1)	(2)	(3)	(4)
Mean_WGI squared	-9.11***	-1.56***	-8.72***	-3.31***	-8.51**	-34.11***	-9.30**	-23.14***
	(0.74)	(0.60)	(0.58)	(0.44)	(4.11)	(1.24)	(4.39)	(1.97)
Mean_WGI	15.36***	0.00	11.69***	0.00	15.56	91.20***	13.79	56.64***
	(1.69)	(.)	(1.34)	(.)	(9.75)	(3.93)	(10.49)	(5.97)
Hospitalizations	4.38***	5.62***	2.00***	3.37***	5.59***	5.62***	3.35***	3.37***
	(0.15)	(0.18)	(0.15)	(0.18)	(1.35)	(1.37)	(1.20)	(1.21)
GDP per capita	7.80***	9.52***	9.45***	9.85***	7.06	-21.66***	9.10**	-17.83***
	(0.85)	(1.57)	(0.68)	(1.16)	(5.29)	(0.06)	(4.48)	(0.70)
Population	2.85***	1.83***	3.24***	2.63***	2.74***	2.61***	3.14***	3.41***
	(0.10)	(0.14)	(0.08)	(0.11)	(0.72)	(0.06)	(0.60)	(0.13)
Population density	-0.08	1.37***	-0.02	1.03***	0.10	0.33***	0.02	-0.06
	(0.12)	(0.17)	(0.10)	(0.13)	(0.84)	(0.03)	(0.73)	(0.13)
Individual fixed effect	No	Yes	No	Yes	No	Yes	No	Yes
Time fixed effect	No	No	Yes	Yes	No	No	Yes	Yes
Observations	9092	9092	9092	9092	9092	9092	9092	9092
R^2	0.17	0.31	0.48	0.60				
R^2_overall					0.17	0.31	0.48	0.60
R^2_between					0.37	1.00	0.46	1.00
R^2_within					0.12	0.12	0.49	0.49

Notes: In all columns I report standard beta coefficients and corresponding significance (* $p < 0.1$, ** $p < 0.05$, *** $p < 0.01$). Heteroskedasticity-robust standard errors in parentheses. Governance quality is measured by Mean_WGI-the mean of all six WGI dimensions. Calculated by Stata 17.0.
